# Cannabis, Religion, and Trust in the Medical Profession: A Cross‐Religious Study of Patients' Attitudes Toward Medical and Recreational Use in Northern Israel

**DOI:** 10.1111/nin.70093

**Published:** 2026-03-15

**Authors:** Loay Zaknoun, Salman Zarka, Ygal Plakht, Orli Grinstein‐Cohen

**Affiliations:** ^1^ Department of Nursing, Faculty of Health Sciences Ben‐Gurion University of the Negev Beer‐Sheva Israel; ^2^ Ziv Medical Center Safed Israel; ^3^ Nursing Department Ben‐Gurion University of the Negev Beer‐Sheva Israel

**Keywords:** cannabis, multi‐faith society, patient attitudes, religion, trust in the medical profession

## Abstract

Despite the global expansion of medical cannabis, limited empirical attention has been given to the sociocultural and religious factors shaping patient attitudes, particularly in multi‐faith societies. Israel provides a distinctive context for such examination, combining advanced medical cannabis regulation with substantial religious diversity. This cross‐sectional study examined how religious affiliation and trust in the medical profession influence attitudes toward medical and recreational cannabis among patients in Northern Israel. A survey was administered to 374 hospitalized patients from four religious groups—Jewish, Muslim, Christian, and Druze—using validated measures of cannabis‐related attitudes and trust in the medical profession. Exploratory factor analyses supported scale validity and reliability. Analyses included ANCOVA and correlations, controlling for age, religiosity, and prior exposure to medical cannabis. Attitudes toward cannabis differed significantly by religious affiliation. Christian participants reported the most favorable views toward both medical and recreational cannabis, followed by Jewish respondents, while Muslim and Druze participants expressed more conservative attitudes. Contrary to prior literature, higher trust in the medical profession was associated with more negative cannabis attitudes overall. A mixed‐design ANCOVA revealed a significant interaction between religious affiliation, trust level, and cannabis type, indicating religion‐specific patterns in how trust shapes cannabis attitudes. These findings underscore the importance of culturally and religiously informed health communication and policy approaches in pluralistic healthcare settings.

## 1 Background

In recent years, the use of medical cannabis has increased significantly worldwide (United Nations 2022), and over the past two decades cannabis has increasingly shifted from being framed primarily as an illicit substance to being debated internationally in medical, public health, and regulatory terms, alongside substantial policy changes across multiple regions (Rehm and Manthey 2020; United Nations 2022; Wanke et al. 2022). Once viewed largely through a criminal or prohibitionist lens, cannabis is now increasingly recognized for its therapeutic potential, alongside ongoing debates regarding recreational use, with many countries legalizing its medical use and, more recently, decriminalizing or legalizing its recreational use (W. Hall and Lynskey 2020; Pacula and Smart 2017). These evolving legal frameworks have amplified public debate and scientific inquiry into the psychological, cultural, and structural determinants of cannabis attitudes and behaviors (Chiu et al. 2022; Siddiqui et al. 2022; Wanke et al. 2022). However, empirical research remains comparatively limited in sociocultural contexts where religion plays a central role in shaping moral reasoning, health‐related values, and trust in medical authority, particularly in the Middle East (Qatanani et al. 2021).

Israel presents a unique case for examining these dynamics. Despite being a global pioneer in the development, clinical research, and regulation of medical cannabis (Mechoulam 2016), recreational use remains illegal, although enforcement has become increasingly relaxed (Sznitman 2020). This regulatory ambivalence—characterized by institutional medical legitimization alongside persistent moral and social stigma—has created a culturally complex landscape in which cannabis is both medicalized and morally contested (Bottorff et al. 2013; W. Hall and Weier 2015; Reid 2020). Comparable regulatory tensions have been shown to shape perceptions of cannabis‐related legitimacy and risk in other national contexts, even when religion is not examined explicitly, underscoring the importance of broader policy and enforcement environments in structuring public attitudes (Moscrop‐Blake and Leal 2025). Within this context, two factors—religious affiliation and trust in the medical profession—are especially salient in shaping public perceptions and personal decisions about both medical and recreational cannabis use (Borojevic and Söhner 2025; Burdette et al. 2018; Kurtzman and Greene 2022). Within Israel's medical cannabis program, physicians function as key gatekeepers, since cannabis may only be prescribed under strict clinical indications and regulatory oversight (Sznitman 2020).

Religious affiliation is widely recognized as a key determinant of health‐related attitudes and behaviors (Cochrane et al. 2024; Koenig 2012). However, much of this evidence derives from western societies with relatively homogeneous religious cultures, offering limited insight into how distinct religious traditions—each with its own theological and moral frameworks—approach the question of cannabis use. Even fewer studies have addressed these issues within religiously pluralistic countries (Rafei et al. 2023). Recent scholarship has further emphasized that religion should not be conceptualized solely as an individual attribute but also as a collective normative system embedded within specific religious traditions, shaping moral judgments and social meanings related to health and deviance (Petrović et al. 2024). This conceptualization may therefore be particularly relevant for understanding cannabis‐related attitudes in societies where religion functions as a shared cultural and moral reference point rather than merely a personal belief system.

The Northern District of Israel provides an ideal setting to address these gaps. It is home to approximately 9% of the country's Jewish population, 68% of its Arab Christian population, 80% of Druze citizens, nearly 34% of its Muslim residents (Central Bureau of Statistics 2025). While the Jewish majority in Israel often approaches health policy and medical innovation through secular–liberal frameworks, Arab religious minorities—including Muslim, Christian, and Druze communities—may engage with such issues through more collectivist and theologically bounded worldviews (Kozlov et al. 2025; Pew Research Center 2016; Zolotov et al. 2018). Studies conducted in other multiethnic and migrant‐receiving societies suggest that religious identity continues to play a central role in shaping health‐related norms and risk perceptions, even under conditions of modernization and cultural pluralism (Gritsenko et al. 2020). These divergent paradigms have practical implications for cannabis‐related attitudes, particularly regarding its legitimacy as a therapeutic modality (Edelstein et al. 2020; Qatanani et al. 2021; Robinson et al. 2020). As a result, medical cannabis is often viewed not only through biomedical lenses but also as a moral, religious, and cultural dilemma (Burdette et al. 2018).

In an era of increasing global migration, healthcare systems are increasingly shaped by the interplay of medical innovation, regulatory change, and religiously grounded moral frameworks (Choi and Kang 2025; Ingleby 2012). From this perspective, Israel can be viewed as a salient microcosm of a broader global phenomenon, offering insight into how such dynamics inform health‐related meanings and practices within culturally and religiously diverse societies.

National survey data support these disparities. A 2019 report by the Israeli Ministry of Public Security (2019), which examined patterns of cannabis use, accessibility, and public attitudes toward legalization, found that Jewish respondents were more likely than Arab respondents to report having friends who use medical cannabis (15% vs. 7%, respectively), knowing how to obtain cannabis (15% vs. 9%, respectively), and supporting legalization for personal use (33% vs. 18%, respectively). Moreover, the perceived health risk from cannabis was significantly higher among Arabs (30%) than among Jews (16%), suggesting a cautious orientation toward cannabis use shaped by broader social norms and collective values, which may intersect with, but are not limited to, formal religious affiliation. These findings reflect broader challenges observed in multicultural societies, where formal policy, clinical standards, and cultural worldviews are not always aligned (Ingleby 2012; Kirmayer 2012; Napier et al. 2014). Importantly, these tensions and challenges affect not only patients but also healthcare professionals. Kurtzman et al. (2022) found that medical cannabis presents a particularly complicated intersection of science, regulations, and values, placing nurses in a challenging position. Their qualitative study of nurse leaders in the United States revealed the ambiguity and uncertainty healthcare providers face when navigating this space, often in the absence of clear guidelines or educational frameworks. Although religion was not the central focus of that study, it underscores how value‐laden and institutionally ambiguous contexts—often shaped by broader cultural and moral frameworks—can influence professional engagement with medical cannabis. Similarly, recent research among healthcare professionals in Northern Israel found that religious affiliation significantly influenced physicians' and nurses' attitudes toward medical cannabis, suggesting that socioreligious factors operate across the clinical encounter (Zaknoun et al. 2025).

In addition to religion, which has been widely recognized as a determinant of health‐related attitudes and behaviors, trust in the medical profession has been widely recognized as a key determinant of health behavior (M. A. Hall et al. 2002; Blendon et al. 2014). Higher trust in healthcare providers has been associated with greater acceptance of controversial or novel medical treatments, including medical cannabis (Kurtzman and Greene 2022). In Israel, studies have shown that Arab citizens and Jewish immigrants report relatively higher levels of institutional trust in the healthcare system than native‐born Jewish population (Pinchas‐Mizrachi et al. 2020). However, how trust in the medical profession interacts with religious identity to shape attitudes toward medical versus recreational cannabis remains largely unexplored.

Despite the growing public interest in cannabis use, empirical research that concurrently examines religion and trust in the medical profession remains notably limited, both internationally and within the Israeli context. Furthermore, the existing professional literature in Israel often categorizes the population dichotomously into Jews and Arabs, treating Muslim, Christian, and Druze communities as a single undifferentiated category, resulting in limited representativeness. This gap is particularly salient given Israel's religious and cultural diversity, which profoundly shapes health‐related perceptions, attitudes, and behaviors in complex ways.

The present study seeks to address this gap by empirically examining the interplay between religious affiliation and trust in the medical profession in shaping attitudes toward medical and recreational cannabis, among a diverse sample of Jewish, Muslim, Christian, and Druze patients in Northern Israel. To our knowledge, this is the first study to explore this topic in a multicultural Israeli context and to include Christian and Druze patients as distinct religious groups. Specifically, we hypothesize that: (1) There will be differences in attitudes toward medical and recreational cannabis based on religious affiliation; (2) Trust in the medical profession will be positively associated with attitudes toward medical and recreational cannabis; and (3) Religious affiliation and trust in the medical profession will moderate the difference in attitudes toward medical and recreational cannabis. Understanding these dynamics is crucial for developing culturally sensitive health communication strategies and for shaping cannabis policy in religiously diverse societies.

## 2 Methods

### 2.1 Study Setting

A cross‐sectional study was conducted at the Ziv Medical Center in Northern Israel—the sole hospital in the region—that serves a demographically diverse population. Its location in a multicultural area, characterized by a rich mosaic of religious and ethnic communities, combined with its role as the principal referral center for patients throughout the region, makes it a particularly suitable site for examining patients' attitudes toward medical and recreational cannabis in a broadly representative, multi‐faith healthcare setting.

### 2.2 Eligibility for Participation

All adult patients (aged ≥ 18 years) hospitalized in the internal medicine departments and surgical departments (including general surgery, orthopedics, urology, otolaryngology, and ophthalmology) during the study period were eligible to receive the questionnaire. Inclusion criteria required:

- proficiency in Hebrew or Arabic;
- ability to provide written informed consent; and
- absence of cognitive impairment or communication limitations that would prevent the ability to provide informed consent or to meaningfully complete the questionnaire.

Patients admitted to psychiatric, pediatric, or obstetric units were not approached. These exclusions were made on ethical grounds, aiming to avoid undue burden, compromised autonomy, or heightened vulnerability associated with these clinical contexts.

### 2.3 Eligibility for Analysis

For inclusion in the analytic sample, only participants who self‐identified as Jewish, Muslim, Christian, or Druze were retained. Participants who selected “other” as their religious affiliation (*n* = 4) were excluded, as this category did not align with the predefined conceptual focus of the study.

### 2.4 Sampling and Recruitment Procedures

A non‐random consecutive sampling strategy was employed. A trained member of the research team approached eligible patients consecutively at their bedside across all participating units. Each potential participant received a standardized verbal explanation detailing the study's aims, procedures, voluntary nature, and confidentiality safeguards. Patients who agreed to participate provided written informed consent in their preferred language (Hebrew or Arabic).

To minimize social desirability bias and reduce potential influence from the research team, the researcher did not remain present during questionnaire completion. Although non‐random, this sampling approach is commonly used in hospital‐based survey research and was selected to maximize feasibility and coverage across eligible clinical units.

### 2.5 Translation and Pilot Testing

The questionnaires were originally developed in English and subsequently translated into Hebrew and Arabic using a standardized forward–backward translation procedure. Two independent bilingual translators translated the questionnaire from English into Hebrew and Arabic, followed by back‐translation into English to ensure semantic, cultural, and conceptual equivalence. Any discrepancies were resolved through consensus discussion among the translators and the research team.

A pilot test involving 10 hospitalized patients was conducted solely to assess the clarity, comprehensibility, and cultural appropriateness of the questionnaire. As no modifications were required, data from the pilot phase were not included in the final analysis, and the instrument was administered unchanged during full data collection.

### 2.6 Data Collection Procedures

Data collection took place between October 2024 and April 2025. All procedures were performed in compliance with relevant laws and institutional guidelines and were approved by the institutional ethics committee (ZIV0037‐24). The researcher explained the study's objectives to the patients, emphasized its anonymity, explained the concept of informed consent, and highlighted the voluntary nature of participation. Written informed consent was obtained from patients prior to data collection, and then the research questionnaires were distributed. To preserve anonymity and further reduce potential social desirability bias—particularly given the sensitivity of topics related to religion and cannabis use—participants were instructed to complete the questionnaire independently and deposit it in a sealed collection box located at the nurses' station. Completed questionnaires were collected daily by the research team and screened for eligibility, completeness, and internal consistency. No identifying information was recorded at any stage of data collection or analysis. A total of 550 questionnaires were distributed, of which 374 were returned, yielding a participation rate of 68%.

### 2.7 Measures

#### 2.7.1 Attitudes Toward Medical and Recreational Cannabis

Attitudes toward cannabis were assessed using a 12‐item self‐report scale adapted from Arora et al. (2020). The scale was adapted to explicitly distinguish between attitudes toward medical and recreational cannabis and to ensure relevance to the Israeli regulatory and sociocultural context, while preserving the original conceptual structure. It included two subcomponents: six items measuring attitudes toward medical cannabis (e.g., “Medical cannabis should be legalized” and “Medical cannabis use is risky”), and six items assessing attitudes toward recreational cannabis (e.g., “Recreational cannabis use is acceptable among adults” and “Recreational cannabis leads to harder drugs”). Participants rated each item on a five‐point Likert scale (from 1 = strongly disagree, to 5 = strongly agree). Total scores range between 6 and 30 for each subcomponent, with higher scores indicating more favorable attitudes toward cannabis use. An exploratory factor analysis (EFA) was conducted using principal component extraction with oblimin rotation on the 12 items. The decision to retain two factors was supported by multiple criteria, including Kaiser's criterion (eigenvalues > 1) (Kaiser 1960), the scree plot, parallel analysis, and Velicer's minimum average partial test (O'Connor 2000). These two factors accounted for approximately 83.5% of the total variance. Item loadings ranged from 0.64 to 0.98, indicating strong associations with their respective factors. The first factor appeared to capture attitudes toward medical cannabis, while the second represented attitudes toward recreational use. Internal consistency for both factors was excellent (see Table 1), and the factors were positively correlated, *r*(372) = 0.56, *p* < 0.001.

**Table 1:** Results of exploratory factor analysis on the attitudes about medical and recreational cannabis items.

Item	Medical cannabis	Recreational cannabis
*Medical cannabis use*		
In favor of legalization	0.98	
Acceptable now	0.97	
Risky (R)	0.97	
Leads to the use of harder drugs (R)	0.96	
Important people in favor	0.93	
Acceptable when younger	0.64	
*Recreational cannabis use*		
In favor of legalization		0.96
Acceptable now		0.96
Risky (R)		0.90
Leads to the use of harder drugs (R)		0.88
Important people in favor		0.86
Acceptable when younger		0.85
Eigenvalue	7.78	2.24
% of variance explained	64.8%	18.7%
Cronbach's *α*	0.96	0.95

*Note: N* = 373–374. Factor loadings above 0.20 are shown.

Abbreviation: R = Reverse scored.

#### 2.7.2 Trust in the Medical Profession

Trust in the medical profession was assessed using the 11‐item scale developed by M. A. Hall et al. (2002). Sample items included statements such as, “Doctors can be trusted to tell the truth about health issues” and “I trust doctors to put patients' needs above all else.” Participants responded using a five‐point Likert scale (from 1 = strongly disagree, to 5 = strongly agree). Total scores range between 11 and 55, with higher scores reflecting greater trust in the medical profession. An EFA using principal component extraction was performed on the 11 items. The decision to retain a single factor was supported by several criteria, including Kaiser's criterion (eigenvalues > 1) (Kaiser 1960), the scree plot, parallel analysis, and Velicer's minimum average partial test (O'Connor 2000). The single factor accounted for approximately 60.2% of the total variance. Item loadings ranged from 0.66 to 0.89, suggesting strong item–factor associations. Internal consistency was excellent (Cronbach's *α* = 0.93).

### 2.8 Data Analysis

The preliminary analyses involved examining differences in background variables across religious affiliation. One‐way analyses of variance (ANOVAs) with Bonferroni‐adjusted post hoc comparisons were used for continuous variables, while chi‐square tests of independence were applied to categorical variables; significant chi‐square results were followed by Bonferroni‐adjusted post hoc tests. Background variables found to differ significantly by religious affiliation were statistically controlled in subsequent hypothesis testing. To evaluate Hypothesis 1, one‐way analyses of covariance (ANCOVAs) with Bonferroni‐adjusted post hoc tests were conducted to compare attitudes toward medical and recreational cannabis across religious groups. For Hypothesis 2, Pearson correlation coefficients were computed to assess associations between trust in the medical profession and attitudes toward both types of cannabis use. Hypothesis 3 was tested using a three‐way mixed‐design ANCOVA, followed by Bonferroni‐adjusted post hoc analyses, to examine differences between medical and recreational cannabis attitudes as a function of trust in the medical profession and religious affiliation. All analyses were performed using IBM SPSS statistics version 30, with a significance threshold of *α* = 0.05 (two‐tailed).

## 3 Results

### 3.1 Preliminary Analysis

Sample characteristics by religious affiliation are presented in Table 2. The distribution of religious affiliations in the sample is consistent with the heterogeneous, multi‐faith population served by the medical center in Northern Israel. Significant differences emerged across age, level of religiosity, and medical cannabis usage by family or friends. Jewish participants were older than their Muslim and Druze counterparts and were more likely to identify as secular than others. Additionally, Jewish participants more frequently reported that a family member or friend had used medical cannabis, relative to Muslim participants.

**Table 2:** Sample characteristics, by the group of religious affiliation.

		Religious affiliation				
	Total	Jewish	Muslim	Christian	Druze	
Variable	(*N* = 374)	(*n* = 141)	(*n* = 77)	(*n* = 67)	(*n* = 89)	Difference test
Age, *M* (SD)	50.36 (17.89)	54.05_a_ (17.80)	47.08_b_ (17.44)	49.99_ab_ (17.98)	47.39_b_ (18.03)	*F*(3, 370) = 3.76, *p* = 0.011, *η* ^2^ = 0.03
Gender, *n* (%)						*χ* ^2^(3, *N* = 374) = 0.83, *p* = 0.842
Male	205 (54.8)	81 (57.4)	40 (51.9)	35 (52.2)	49 (55.1)	
Female	169 (45.2)	60 (42.6)	37 (48.1)	32 (47.8)	40 (44.9)	
Marital status, *n* (%)						*χ* ^2^(3, *N* = 374) = 0.82, *p* = 0.844
Married or in a relationship	271 (72.5)	99 (70.2)	56 (72.7)	51 (76.1)	65 (73.0)	
Other	103 (27.5)	42 (29.8)	21 (27.3)	16 (23.9)	24 (27.0)	
Religiosity, *n* (%)						*χ* ^2^(3, *N* = 374) = 70.77, *p* < 0.001
Secular	61 (16.3)	52_a_ (36.9)	1_b_ (1.3)	4_b_ (6.0)	4_b_ (4.5)	
Any degree of religiosity	313 (83.7)	89_a_ (63.1)	76_b_ (98.7)	63_b_ (94.0)	85_b_ (95.5)	
Education, *n* (%)						*χ* ^2^(3, *N* = 374) = 7.78, *p* = 0.051
Not academic.	239 (63.9)	93 (66.0)	53 (68.8)	33 (49.3)	60 (67.4)	
Academic	135 (36.1)	48 (34.0)	24 (31.2)	34 (50.7)	29 (32.6)	
Participant with an oncological disease						*χ* ^2^(3, *N* = 374) = 7.20, *p* = 0.066
Yes (active/remission/recovered)	32 (8.6)	17 (12.1)	3 (3.9)	8 (11.9)	4 (4.5)	
No	342 (91.4)	124 (87.9)	74 (96.1)	59 (88.1)	85 (95.5)	
Family member(s) or friend(s) with an oncological disease						*χ* ^2^(3, *N* = 374) = 3.48, *p* = 0.323
Yes (active/remission/recovered)	125 (33.4)	49 (34.8)	20 (26.0)	27 (40.3)	29 (32.6)	
No	249 (66.6)	92 (65.2)	57 (74.0)	40 (59.7)	60 (67.4)	
Family member(s) or friend(s) who use medical cannabis, *n* (%)						*χ* ^2^(3, *N* = 374) = 13.20, *p* = 0.004
Yes	105 (28.1)	54_a_ (38.3)	13_b_ (16.9)	17_a,b_ (25.4)	21_ab_ (23.6)	
No	269 (71.9)	87_a_ (61.7)	64_b_ (83.1)	50_ab_ (74.6)	68_ab_ (76.4)	
Family member(s) or friend(s) who use recreational cannabis, *n* (%)						*χ* ^2^(3, *N* = 374) = 5.56, *p* = 0.135
Yes	68 (18.2)	27 (19.1)	8 (10.4)	17 (25.4)	16 (18.0)	
No	306 (81.8)	114 (80.9)	69 (89.6)	50 (74.6)	73 (82.0)	

*Note:* Categories with different subscript letters differ significantly from each other at the 0.05 level.

### 3.2 Hypothesis Testing

Table 3 summarizes comparisons of attitudes toward medical and recreational cannabis across religious affiliations. Supporting Hypothesis 1, ANCOVAs revealed significant results, suggesting that Christians scored significantly higher than all other groups on both medical and recreational cannabis. Jewish participants also scored significantly higher than Muslim and Druze participants, who did not differ significantly from each other.

**Table 3:** Means, adjusted means, standard deviations, and one‐way ANCOVA in measures of attitudes toward medical and recreational cannabis.

		Religious affiliation				
	Total sample	Jewish	Muslim	Christian	Druze	
	(*N* = 374)	(*n* = 141)	(*n* = 77)	(*n* = 67)	(*n* = 89)	
Variable	*M*/Adj. *M* (SD)	*M*/Adj. *M* (SD)	*M*/Adj. *M* (SD)	*M*/Adj. *M* (SD)	*M*/Adj. *M* (SD)	Difference test
Medical cannabis	16.27/16.02 (8.81)	20.39/19.60_a_ (7.10)	9.27/10.02_b_ (6.14)	23.28/23.63_c_ (5.80)	10.51/10.85_b_ (7.06)	*F*(3, 367) = 89.94, *p* < 0.001, *η* ^2^_*p*_ = 0.42
Recreational cannabis	9.02/9.10 (5.45)	9.96/9.55_a_ (5.98)	6.69/7.03_b_ (2.82)	12.31/12.57_c_ (6.34)	7.08/7.24_b_ (3.70)	*F*(3, 367) = 20.85, *p* < 0.001, *η* ^2^_*p*_ = 0.15

*Note:* All results are controlled for age, religiosity level, and medical cannabis usage by family members or friends. Attitude scores represent summed responses on six‐item scales ranging from 6 to 30, with higher scores indicating more favorable attitudes toward medical or recreational cannabis.

Abbreviation: Adj. = adjusted.

Table 4 presents correlations between trust in the medical profession and cannabis attitudes, both in the overall sample and stratified by religious affiliation. Contrary to Hypothesis 2, trust in the medical profession was significantly negatively associated with attitudes toward medical cannabis in the total sample, as well as among Muslim and Druze participants. Trust was also significantly negatively related to attitudes toward recreational cannabis in the total sample and among Christians.

**Table 4:** Associations between trust in medical profession and measures of attitudes toward medical and recreational cannabis.

		Religious affiliation			
	Total sample	Jewish	Muslim	Christian	Druze
Variable	(*N* = 374)	(*n* = 141)	(*n* = 77)	(*n* = 67)	(*n* = 89)
Medical cannabis	−0.38***	0.04	−0.27*	−0.23	−0.42***
Recreational cannabis	−0.24***	−0.08	−0.09	−0.30*	−0.18

* *p* < 0.05

*** *p* < 0.001.

**Figure 1:**
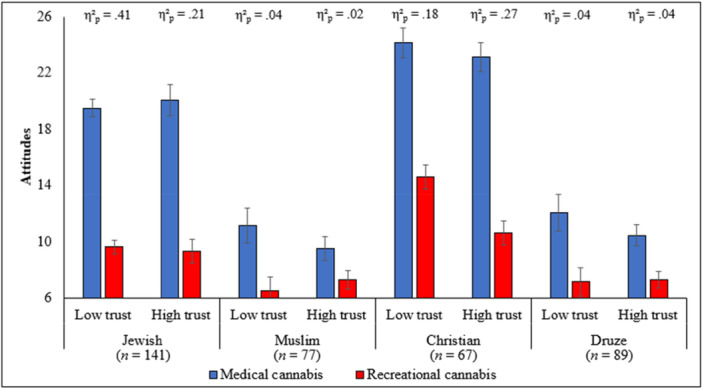
Means and standard errors of attitudes toward cannabis by religious affiliation, trust in medical profession, and type of cannabis. Note. All results are controlled for age, religiosity level, and medical cannabis usage by family members or friends.

A three‐way mixed‐design ANCOVA showed a significant interaction between religious affiliation, trust in the medical profession, and cannabis type (medical vs. recreational), *F*(3, 363) = 4.18, *p* = 0.006, *η* ^2^_*p*_ = 0.03, providing support for Hypothesis 3. To facilitate interpretation, trust in the medical profession was dichotomized at the median (*Med* = 42.50) to ensure adequate group sizes. Post hoc analyses indicated that, across all groups and both trust levels (low vs. high), medical cannabis was rated more favorably than recreational cannabis (all *p*s < 0.012). However, this difference was more pronounced among Jewish and Christian participants compared to Muslims and Druze, as indicated by larger effect sizes (see Figure 1). Notably, among Jewish participants, the disparity between medical and recreational attitudes was greater in the low‐trust group than in the high‐trust group, whereas among Christians, the disparity was greater in the high‐trust group compared to the low‐trust group. For Muslims and Druze, this difference was similar across both trust levels.

## 4 Discussion

This study advances understanding of how religious affiliation and trust in the medical profession jointly shape attitudes toward medical and recreational cannabis among patients from diverse religious communities. Consistent with the study hypotheses, the findings—illustrated in Figure 1—demonstrate that trust does not exert a uniform influence across religious groups, but rather interacts with religious affiliation to differentially shape attitudes toward medical and recreational cannabis. The findings offer empirical support for the hypothesis that cannabis‐related attitudes are shaped not only by biomedical knowledge or regulatory frameworks, but also by socioreligious worldviews and trust in the medical profession. Together, these factors appear to inform how medical legitimacy, perceived risk, and the acceptability of medical and recreational cannabis use are interpreted across different religious communities.

By examining distinct religious groups within the same healthcare system, the study demonstrates that attitudes toward medical and recreational cannabis are not uniform, but rather reflect religion‐specific moral logics, theological traditions, and differentiated relationships with medical authority. The observed variation—where Christian participants expressed the most favorable views toward both medical and recreational cannabis, followed by Jewish respondents, while Muslim and Druze participants adopted more conservative and restrictive attitudes—directly corresponds to the interaction patterns shown in Figure 1, particularly the differing effects of high versus low trust within each religious group. These findings highlight how contested medical interventions are negotiated at the intersection of faith‐based moral frameworks and trust in the medical profession.

### 4.1 Religious Affiliation and Cannabis Attitudes

Religion remains a central determinant of health‐related beliefs and behaviors (Cochrane et al. 2024; Koenig 2012). Our findings align with prior literature emphasizing the influential role of religion in shaping individuals' attitudes toward controlled substances, including cannabis (Burdette et al. 2018). Notably, Christian participants in our sample demonstrated the most favorable attitudes toward medical cannabis. This trend may reflect a convergence of theological doctrines and religiously embedded cultural narratives within Christian traditions that emphasize compassion, palliative care, and the ethical imperative to alleviate suffering (Ferrara 2021).

The Catechism of the Catholic Church (n.d.) discourages non‐medical drug use but permits therapeutic applications under medical supervision, reflecting a broader ethical commitment to reduce human suffering and promoting compassion. This doctrinal stance can be interpreted as providing moral space for the acceptance of medical cannabis within clinical or palliative contexts, as Christian ethical frameworks emphasize compassion, the alleviation of suffering, and the moral evaluation of medical interventions in healthcare decision‐making (Koenig 2012). As Ferrara (2021) notes, cannabis has historically been integrated into Christian mystical and folk healing traditions, where it was used to facilitate spiritual insight or healing rituals. Such historical associations may provide a symbolic interpretive framework, through which contemporary Christian patients may interpret and morally justify the use of medical cannabis. Furthermore, ethnobotanical evidence highlights the longstanding use of cannabis in spiritual and therapeutic settings across western traditions, including Christian contexts (Balant et al. 2021).

While contemporary Christian patients may not be explicitly aware of the historical or ethnobotanical associations with cannabis, such narratives may nonetheless operate as religiously embedded cultural meanings that can shape moral intuitions implicitly rather than through conscious doctrinal knowledge. Accordingly, Christian respondents may have been more receptive to medical cannabis insofar as it aligns with values of healing and compassion, rather than conflicting with religious morality. Notably, favorable attitudes toward recreational cannabis among Christian participants suggest a comparatively broader permissiveness that extends beyond strictly therapeutic contexts, a pattern that may reflect wider cultural or social influences rather than religiously grounded moral reasoning.

It should be noted that although the Jewish subgroup in the present sample was predominantly religious, it was comparatively more secular than the other religious groups included in the study (see Table 2). Jewish participants expressed moderately favorable attitudes toward medical cannabis, while exhibiting greater caution regarding its recreational use. This pattern is consistent with longstanding tensions within Jewish legal and ethical frameworks, which generally prohibit recreational intoxication but permit medical interventions under the doctrine of *pikuach nefesh*—the imperative to preserve life that overrides most religious prohibitions (Liberman 2024; Davis 2007). Accordingly, medical cannabis may be viewed within Jewish religious discourse as a legitimate therapeutic intervention rather than a morally problematic substance. Leading rabbinic authorities have endorsed the use of medical cannabis in cases of chronic pain or severe illness, framing it as both a halachic (Jewish law) allowance and, in some cases, as a moral obligation to alleviate suffering (Bleich 2022). This religious legitimation may contribute to the relatively favorable attitudes observed toward medical cannabis among Jewish participants in the present study. At the same time, the presence of favorable attitudes toward recreational cannabis among Jewish participants suggests a degree of openness that extends beyond strictly halachic considerations, potentially reflecting broader cultural or social influences rather than adherence to religious norms alone.

Historical perspectives have also been discussed in relation to cannabis within Jewish tradition. Archeological and interpretive scholarship has suggested that cannabis may have been present in ancient Israelite ritual contexts, potentially identified with Kaneh‐bosem—a component of the biblical anointing oil described in Exodus 30:23 (Benet, 1936 as cited by Pisanti and Bifulco 2019). While this identification remains debated within biblical and ethnobotanical scholarship, it may serve as a symbolic interpretive backdrop through which cannabis can be understood as compatible with religious tradition when framed as a therapeutic and life‐preserving intervention.

Additionally, sociocultural factors may further shape contemporary attitudes toward cannabis within Jewish communities. Secular Jewish populations in Israel often hold more permissive views, likely influenced by cannabis normalization in local media and public discourse (Kozlov et al. 2025). By contrast, Ultra‐Orthodox communities tend to maintain more conservative attitudes, particularly toward recreational use, guided by rabbinic authority and communal norms (Serfaty et al. 2021). This internal diversity highlights that the influence of public discourse is not uniform across religious subgroups but may be mediated by differing levels of religious authority, community structure, and engagement with mainstream cultural narratives. Nevertheless, across these varying religious contexts, medical cannabis is increasingly framed across religious Jewish communities as compatible with halachic values when situated within a clearly defined framework of medical necessity and the preservation of life.

Muslim participants in our study consistently exhibited conservative attitudes toward both medical and recreational cannabis use. Such views align with dominant interpretations within Islamic jurisprudence, which classify intoxicants as *haram* (forbidden), based on Quranic verses such as *Surah al‐Ma'idah* 5:90, which denounce intoxicants as “abominations and the work of Satan” (The Qur'an, trans. Abdel Haleem 2020). While cannabis is not explicitly mentioned in the *Qur'an* or the *Hadith* (the body of traditions and sayings attributed to the Prophet Muhammad), many classical and contemporary Islamic scholars have historically analogized it to alcohol due to its psychoactive properties, thereby extending the prohibition to cannabis use (Ghiabi 2019; Qatanani et al. 2021).

Nonetheless, Islamic legal doctrine includes the principle of *zarurat* (necessity), which permits otherwise forbidden substances when required to preserve life or relieve suffering. Under this doctrine, several modern *fatwas* (religious legal rulings) have endorsed the use of medical cannabis in cases of severe illness or chronic pain (Siddiqui et al. 2022). However, the presence of such doctrinal permissibility does not necessarily translate into broad social or moral acceptance. In many Muslim communities, cannabis use continues to be associated with a religiously and culturally entrenched stigma, shaped not only by theological interpretation but also by broader sociocultural norms, concerns regarding addiction, and historical associations with recreational drug misuse and moral decline (Ghiabi 2019; Siddiqui et al. 2022). Within this context, high trust in medical authority may, in some contexts, reinforce caution, particularly when cannabis is perceived as a last‐resort or ethically sensitive intervention within the healthcare system. These intersecting religious and cultural dynamics may help explain the pronounced caution observed among Muslim participants in our sample, even in medically sanctioned contexts.

Druze participants demonstrated conservative attitudes toward both medical and recreational cannabis, closely resembling the patterns observed among Muslim respondents. The Druze religion maintains strict doctrinal secrecy. Its sacred texts, *Rasa'il al‐Hikma* (Epistles of Wisdom), are accessible only to the initiated elite (*ʿuqqāl*), while lay members (*juhhāl*) are excluded from religious teachings and interpretation (Firro 1992; Saab 2000). Due to this esoteric structure, no formal or public religious rulings on cannabis use have been articulated.

Nonetheless, Druze religious norms emphasize self‐restraint, mental clarity, and moral discipline—values that may implicitly discourage substance use (Firro 1992; Kheir 2025). These principles are not only spiritual ideals but also shape broader communal expectations for both religious and secular segments of the Druze society (Kheir 2025). Within this context, attitudes toward substances such as cannabis are likely shaped less by explicit doctrinal prohibition and more by communal norms, identity‐based expectations, and moral boundaries embedded within Druze collective life. In this light, the conservative responses observed among Druze participants in the present study may reflect the internalization of communal ethical standards and social norms, rather than adherence to articulated religious rulings regarding cannabis use. Such dynamics may help explain the persistence of cautious attitudes toward both medical and recreational cannabis, even in the absence of formal religious guidance and within medically sanctioned contexts.

### 4.2 Trust in the Medical Profession

At the aggregate level in the present study, higher trust in the medical profession was significantly associated with more negative attitudes toward both medical and recreational cannabis. While earlier research has linked greater trust in the medical profession with increased openness to medical cannabis (Kurtzman and Greene 2022), the present findings indicate that this association is not uniform and may be contingent upon religious affiliation. In this sense, the overall negative association observed in the total sample masks substantial heterogeneity across religious groups, revealing divergent trust‐related patterns when examined within specific socioreligious contexts.

Specifically, among Muslim and Druze participants, higher trust in the medical profession was significantly and inversely associated with attitudes toward medical cannabis, with the strongest effect observed among Druze respondents. Importantly, this inverse association did not extend to recreational cannabis, suggesting that trust‐related restraint operates primarily within the medical domain. In these religiously conservative communities, trust in the medical profession may reinforce caution rather than permissiveness, whereby individuals who express confidence in the medical profession align more closely with prevailing clinical norms that position medical cannabis as a tightly regulated or last‐resort intervention. This interpretation is consistent with the highly controlled nature of Israel's medical cannabis program and the documented reluctance among physicians to prescribe cannabis except under specific clinical conditions (Sznitman 2020).

Among Christian participants, trust in the medical profession was negatively associated with attitudes toward recreational cannabis, while no significant association was observed with attitudes toward medical cannabis. Although Christian participants in the present sample also reported high levels of religiosity, this pattern suggests a differentiation between medically sanctioned therapeutic use and morally charged leisure consumption, whereby trust may function as a boundary‐setting mechanism that constrains acceptance of recreational use without diminishing openness to clinically legitimized interventions.

In contrast, no statistically significant associations between trust and attitudes toward either medical or recreational cannabis were observed among Jewish participants. This absence of a consistent trust‐related pattern may reflect the considerable heterogeneity within Israeli Jewish society, encompassing a wide spectrum of religiosity, cultural orientations, and relationships with medical authority, as well as the coexistence of secular and religious health paradigms (Koenig 2012; Sznitman 2020).

Taken together, these findings demonstrate that trust in the medical profession does not uniformly promote acceptance of cannabis‐related interventions. Rather, its influence is context‐dependent and shaped by religious affiliation and moral meanings attached to cannabis use, functioning in some settings as a mechanism of restraint and in others as a normative filter distinguishing between therapeutic legitimacy and morally contested leisure practices.

## 5 Policy and Practice Implications

The findings highlight the need for culturally and religiously tailored health communication strategies when addressing medical cannabis in multi‐faith healthcare settings such as Northern Israel. Importantly, the results demonstrate that trust in the medical profession does not function uniformly as a facilitator of acceptance. Rather, its influence varies by religious affiliation and by the moral framing of cannabis use, underscoring the limitations of one‐size‐fits‐all policy and communication approaches. These findings have implications not only for relationships between patients and healthcare professionals, but also for trust in healthcare systems more broadly. At the system level, trust is shaped through institutional practices, consistency of messaging, and the coherence of medical guidance across professional roles.

In Muslim and Druze communities, where higher trust in the medical profession was associated with more cautious attitudes toward medical cannabis, efforts to simply “strengthen trust” may be insufficient or even counterproductive. Instead, effective engagement may require explicitly addressing the moral and ethical boundaries through which medical cannabis is interpreted. Collaboration with trusted religious and community figures can help reframe cannabis as a medically sanctioned, necessity‐based intervention rather than an illicit or morally ambiguous substance, drawing on concepts such as *zarurat* (necessity) in Islam or Druze ethical emphases on self‐discipline and preservation of health.

Among Jewish and Christian populations, where acceptance of medical cannabis appears more closely aligned with ethical doctrines emphasizing compassion, healing, and life preservation, collaboration with rabbis and clergy may support framing medical cannabis within established moral frameworks such as *pikuach nefesh* in Judaism or principles of healing and compassion in Christian ethics. At the same time, the differentiation observed between medical and recreational cannabis—particularly among Christian participants—suggests that policies and educational initiatives should clearly distinguish between therapeutic legitimacy and leisure use, rather than addressing cannabis as a single, undifferentiated category.

Across all groups, the findings point to the importance of religious literacy and cultural competence training for healthcare providers. Such training can equip providers to recognize when trust may operate as a mechanism of restraint rather than openness and to adapt their communication accordingly. For nurses and other frontline healthcare professionals, who often provide continuity of care and serve as primary points of contact within healthcare systems, trust is constructed through everyday interactions, institutional norms, and the perceived alignment between policy and practice. These approaches are not intended to replace clinical judgment, but to facilitate more effective, ethically sensitive dialogue around contested treatments, thereby improving alignment between medical recommendations and patients' moral worldviews.

Beyond the Northern Israeli context, these insights are relevant for healthcare systems serving religiously diverse populations worldwide. As access to medical cannabis expands globally, policies and clinical practices must account for the ways in which religious identity and trust in the medical profession jointly shape patient responses. Strengthening trust may therefore require system‐level transparency, interprofessional alignment, and culturally informed institutional communication, rather than a sole focus on individual clinicians. Bridging the gap between biomedical innovation and faith‐based moral reasoning—through context‐sensitive communication and collaboration—may contribute to reducing stigma, promoting equitable access to care, and enhancing patient engagement in culturally pluralistic societies.

## 6 Limitations and Future Research

While the study offers meaningful insights into the interplay between religion, trust in the medical profession, and attitudes toward medical cannabis, it has several limitations that warrant consideration. Its cross‐sectional design precludes causal inference, and the reliance on self‐reported data may introduce response biases, including the social desirability bias, particularly among participants from conservative religious backgrounds. Moreover, the study was conducted at a single medical center located in a peripheral region of Israel, where sociocultural and religious dynamics may differ from those in other parts of the country, especially in central areas. Replicating the study across diverse geographic regions would enhance its generalizability and provide a more comprehensive national perspective.

Future research should incorporate qualitative methods—such as in‐depth interviews with patients, healthcare providers, and religious leaders—to explore the theological, cultural, and institutional frameworks that shape cannabis‐related attitudes. Further studies are needed to examine intra‐religious variation within religious groups, investigate behavioral outcomes beyond stated attitudes, and assess how evolving medical policies and religious narratives interact to influence attitudes toward medical cannabis over time. Ultimately, such research would contribute to the development of culturally sensitive and religiously informed health communication strategies and cannabis policy. These approaches would be particularly valuable in multi‐faith societies, where religious identity, moral reasoning, and trust in the medical profession play a central role in shaping health‐related decision‐making. As healthcare becomes increasingly multicultural, bridging these domains is essential for promoting both clinical efficacy and patient trust in the medical profession.

## 7 Conclusions and Recommendations

This study underscores the significant role of religious affiliation and trust in the medical profession in shaping patients' attitudes toward medical and recreational cannabis use within a multi‐faith healthcare context. The findings demonstrate that such attitudes are not determined solely by biomedical evidence or regulatory frameworks, but are also deeply shaped by religious worldviews, the moral values they promote, and trust in the medical profession. Importantly, the results indicate that trust in the medical profession does not function uniformly as a facilitator of acceptance, but may operate differently across religious groups, sometimes reinforcing openness and in other cases amplifying restraint.

To ensure equitable and religiously sensitive integration of medical cannabis, healthcare systems may need to move beyond one‐size‐fits‐all approaches. Public health strategies can benefit from being designed with attention to religious diversity, including engagement with trusted religious leaders and tailoring messages to align with patients' ethical frameworks. Enhancing religious competence among healthcare providers and fostering dialogue between medical and religious institutions are essential for reducing stigma, improving access, and strengthening trust in the medical profession, particularly in pluralistic societies where faith and medicine frequently intersect. In this context, strengthening trust should not be understood as uniformly increasing acceptance of medical cannabis, but rather as promoting transparent, religiously informed communication that enables patients to interpret medical recommendations within their moral and religious frameworks. Such an approach may also enhance education of patients and healthcare providers regarding medical cannabis, clarifying its therapeutic role, regulatory boundaries, and ethical positioning across different religious contexts.

## Author Contributions

Conceptualization: Y.P. and O.G.‐C. Data curation: L.Z. Formal analysis: L.Z. and O.G.‐C. Investigation: Y.P. and O.G.‐C. Supervision: S.Z. and O.G.‐C. Writing – original draft: L.Z. Writing – review and editing: Y.P., S.Z., and O.G.‐C. All authors have read and agreed to the published version of the manuscript.

## Funding

The authors received no specific funding for this work.

## Ethics Statement

Ethical approval was received from the institutional ethics committee (ZIV‐0037‐24) on 16 May 2024.

## Consent

All participants signed an informed consent form.

## Conflicts of Interest

The authors declare no conflicts of interest.

## Data Availability

The data that support the findings of this study are available upon request from the corresponding author.
